# Does Health Consciousness Matter to Adopt New Technology? An Integrated Model of UTAUT2 With SEM-*f*sQCA Approach

**DOI:** 10.3389/fpsyg.2022.836194

**Published:** 2022-02-10

**Authors:** Sohaib Mustafa, Wen Zhang, Muhammad Usman Shehzad, Aliya Anwar, Gelas Rubakula

**Affiliations:** ^1^College of Economics and Management, Beijing University of Technology, Beijing, China; ^2^School of Management Engineering, Zhengzhou University, Zhengzhou, China; ^3^School of Economics and Management, North China Electric Power University, Beijing, China; ^4^Institute of Development Studies, University of Dar es Salaam, Dar es Salaam, Tanzania

**Keywords:** 5G technology adoption, structural equation modeling (SEM), fuzzy set qualitative comparative analysis (*fs*QCA), UTAUT2, health-consciousness attitude, behavioral intention

## Abstract

Every emerging technology has its pros and cons; health-conscious users pay more importance to healthy and environment-friendly technologies. Based on the UTAUT2 model, we proposed a comprehensive novel model to study the factors influencing consumers’ decision-making to adopt the technology. Compared to prior studies that focused on linear models to investigate consumers’ technology adoption intentions and use behavior. This study used a Structural Equation Modeling-fuzzy set qualitative comparative analysis (SEM-*fs*QCA) approach to account for the complexity of customers’ decision-making processes in adopting new technology. We collected valid responses from 830 consumers, analyzed them, and evaluated them using a deep learning SEM-*fs*QCA technique to capture symmetric and asymmetric relations between variables. We have extensively incorporated a health-consciousness attitude as a predictor and mediator to understand better the decision-making toward technology adoption, specifically 5G technology. All the factors tested in our model are statistically significant except the economic factors. Health-consciousness attitude (HCA) and behavioral intention (BI) found significant predictors and valid mediators in the process of 5G technology adoption. *F*sQCA provided six configurations to achieve high 5G adoption. The findings have significant practical ramifications for telecom corporations, advertisers, government officials, and key policymakers. Additionally, the study added substantial theoretical literature to technology adoption, particularly the adoption of 5G technology.

## Introduction

Every innovation and technology comes up with its positive and negative aspects. Some innovations/technologies are eco-friendly, while others cause unrecoverable damage to the ecosystem and human health. Human perception about the new technology plays a significant role in its adoption or rejection. Solar heaters, Electronic vehicles, smart homes, and waste-to-energy are some common examples of eco-friendly technologies. These technologies are not only cost-efficient but also protect the ecological system. Opinions on cellular technology, in particular, are divided. Some see them as eco-friendly, while others are concerned about the radiation used to stimulate the signals from these devices.

Since the first analog devices in the 1980s, digital communication technology has evolved dramatically. Both 2G and 3G improved cell phone service coverage, but the third generation introduced mobile internet (Siau and Shen, 2003). Fourth-generation (4G) wireless networks allow banking, retail, and entertainment industries to operate more efficiently (Hasan and Islam, 2014). The ultra-high-speed of 5G has the potential to redefine the versatility of a cell phone. The next generation of wireless broadband is expected to deliver multi-Mbps peak data speeds, ultra-low latency, significant network bandwidth improvements, and increased connection quality stability across a wide range of users. Enhances the user’s overall productivity, creates new opportunities, and connects previously unconnected industries (Qualcomm, 2021).

Human society is expected to demand 5G mobile broadband, resulting in an interconnected multi-source and multi-technology mobile connectivity system capable of meeting future requirements, including technological evolution and advancements in networking technologies (Chen and Yuan, 2016). By the end of 2025, it is estimated that about 37.1 million to 44.6 million people will have adopted 5G broadband (Jahng and Park, 2020). For the time being, 5G is deploying faster than 4G. 4G adoption reached 696,404 users 4 months after commercialization, but 5G adoption reached 784,215 in 8 weeks (Jahng and Park, 2020). The projected yearly growth rate will be 150% during the initial 6 years (Research, 2020). According to some analysts, since the mobile market is known for its short product life cycles, consumer hesitation may result in non-adoption (Maeng et al., 2020). Without considering the possibility of future demands, future revenues would be overstated. The negative forecasts for the future market impede the spread of 5G technology (Maeng et al., 2020).

Health consciousness attitude (HCA) is an essential factor behind human decision-making to adopt or reject new technology (Nagaraj, 2021). This factor is neglected in the technology adoption models, and previous studies rarely investigate this factor concerning new technology adoption or rejection (Venkatesh et al., 2012; Kim et al., 2018). Specifically, the mediating role of health consciousness is never studied in technology adoption models. Fewer studies have been done on the impact of F&B stores on expediting the mediating effect of health consciousness and service efficiency (Nagaraj, 2021). According to researchers, certain psychological factors influencing decision-making are essential to adopt while examining technology adoption (Nagaraj, 2021).

Furthermore, as the researcher (Venkatesh et al., 2012) suggested integrating more variables in their famous work to understand better technology adoption, we have integrated new variables in UTAUT2 to study technology adoption. We have tested our model on adopting and using emerging 5G technology.

Without question, consumers’ adoption of a new product or technology is influenced by social, economic, intrinsic, and psychological factors. These factors contribute to either a low or a high adoption rate. Regardless of the competitive advantages of 5G, the factors listed in this paragraph would ultimately affect adopters or users of 5G technology. Thus, it is important to conduct empirical research into the factors that affect a user’s decision to adopt a new 5G technology. This will improve our understanding of consumer attitudes toward 5G implementation and assist telecommunications companies in developing sound policies for deploying 5G technology in new markets.

We have used the state-of-the-art two-step SEM-*fs*QCA technique to look at the factors behind the 5G technology adoption. This technique is extensively employed to explore technology adoption and is suggested by numerous scholars (Kaya et al., 2020; Xie and Tsai, 2021). We implied a structural equation model (SEM) to test the presented hypothesis first. After initial test results, we used the *fs*QCA approach to get the different possible configurations of our variables to achieve high 5G technology adoption.

The following research questions are proposed for this study to address the research gap.

RQ 1. How do various factors influence consumers’ decision-making in 5G technology adoption?

RQ 2. Does Health consciousness attitude play any role as a mediator or influential factor behind technology adoption?

RQ 3. What are the different possible combinations of factors to achieve high 5G adoption?

Structural equation modeling results at the first stage presented that Health consciousness attitude (HCA), Intrinsic factors (IF), psychological factors, social factors, and behavioral intention (BI) are statistically significant, except economic factors (EF), which is not statistically significant to 5G technology adoption (TA). Our model explained 77.3, 44.9, and 37% variance at BI, HCA, and TA in SEM results, respectively. In the second step, the fuzzy set qualitative comparative analysis (*fs*QCA) approach provided six different configurations for high technology adoption that yield 82.9% overall solution coverage. We can lead to the maximum 5G technology adoption results by following any given configurations or combining different configurations. Hence, we propose this methodology to predict human decision-making and the asymmetric relationship between variables.

## Theoretical Structure

To better understand user behavior and the factors influencing users’ intentions to use new technology, we suggested an integrated model of Unified Theory of Acceptance and Use of Technology (UTAUT2). The reason behind choosing UTAUT2 is that the original model was also tested on mobile internet adoption, making it a perfect match for our study.

### Unified Theory of Acceptance and Use of Technology

Numerous technology acceptance models have been developed, each with its determinants. Venkatesh et al. (2012) reviewed and compared existing models, developed an adoption theory and validated the empirical theory (Venkatesh et al., 2012). The key idea of our study is to extend and expand the current UTAUT2 model in the context of 5G internet technology usage behavior as suggested by researchers (Venkatesh et al., 2012). UTAUT2 is a modified version of UTAUT, which was designed based on eight SI theories: the TRA, the C-TAM, the MPCU, the IDT, the TAM, the MM, and the TPB. Additional research is needed to elucidate the purpose and behavioral implications of Venkatesh and his colleagues’ UTAUT model. Many researchers have integrated new variables in UTAUT2 and studied different technology adoptions, such as the adoption of public WiFi (Aswani et al., 2018), adoption of smartphone fitness applications (Dhiman et al., 2019), internet banking (Alalwan et al., 2018), mHealth adoption (Alam et al., 2021), and so on. At the same time, HCA is never being studied extensively as a part of any TAM.

Hence, we added HCA in our proposed model and developed a model tailored to 5G internet technology requirements to aid current research. The model postulate to be backed by HCA, intrinsic, psychological, social, and economic influences. Having close personal ties and a creative outlook are critical factors in choosing a useful and efficient tool. This model uncovers the users’ antecedent ideas and behavioral motivations. We agree that human attributes, economic factors, and social factors impact how people perceive innovation. Previous studies in context to usage behavior often ignore the element of *health consciousness attitude* in human nature. Recent researchers have found that the health consciousness nature of humans is a significant factor behind behavioral intention (Shah et al., 2021).

Health awareness was previously established as a proxy for an individual’s inherent drive to preserve good health (Dutta-Bergman, 2005; Choi and Kwon, 2020). Another research discovered a favorable correlation between health consciousness and purchasing decisions (Nagaraj, 2021). According to research on Korean undergraduate students, health consciousness is associated with behavioral intention and behavior (physical activity) (Hong and Chung, 2020). So we believe that consumers have health concerns about new technologies, and health consciousness is a significant factor in adopting new technologies. Hence, we have integrated health consciousness in UTAUT2 and identified its role as a mediator in technology adoption.

Furthermore, unlike the base model, we have merged more influential factors and developed a reflective-formative construct indicating intrinsic, social, psychological, and economic factors.

**FIGURE 1:**
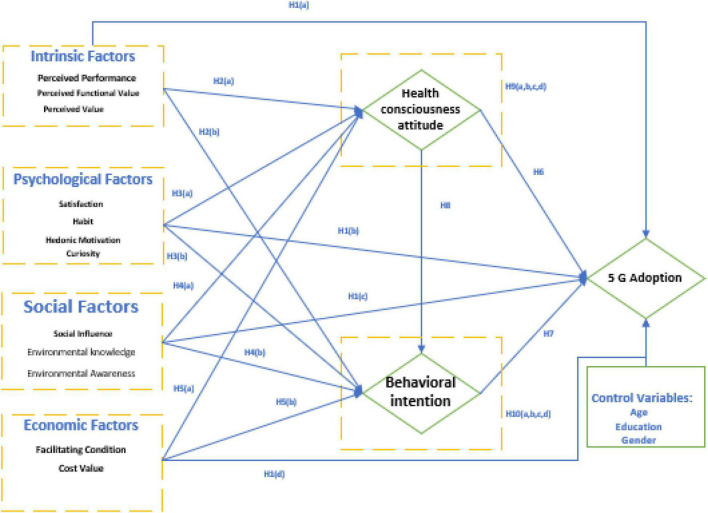
Conceptual framework.

To investigate how users adopt 5G technology and how intrinsic, physiological, social, economic factors, behavioral intention, and Health consciousness attitude influence their decision making. We proposed an integrated model of UTAUT2 (Figure 1).

## Formulation of Hypotheses

### Intrinsic Factors (Perceived Performance, Perceived Functional Value, and Perceived Value), Health Consciousness, Behavioral Intention, and 5G Adoption

In prior research, several intrinsic factors have been identified as important predictors of behavioral intention and adoption – intrinsic means belonging to a thing’s essential nature or constitution (Corduneanu, 2020). Several intrinsic factors were identified and studied in previous studies, such as perceived performance, perceived functional value, and perceived value in technology adoption models. Perceived performance is described as the degree to which a person comprehends that using an information system will aid them in achieving a specific performance objective (Venkatesh et al., 2003). In mobile apps, perceived performance captures behavioral intentions, functional intentions, and adoptions (Chipeva et al., 2018). People are more receptive when new technology is readily available and simple to use (Wu et al., 2021). In cell phone services, perceived performance is an important predictor (Rita et al., 2018). Previous research has also shown that consumers universally accept it when technology or product possesses outstanding functional qualities (Yeh et al., 2016). Perceived value describes as an individual’s assessment of the benefits that specific products or activities can provide (Chen and Lin, 2018). The term “perceived value” refers to consumers’ benefits that 5G technology can offer. As long as the consumer assumes 5G has utility, the assumption is accepted as valid even if they assume the potential has not yet been realized. Specifically, for 5G adoption, perceived value is important (Shah et al., 2021). Thus, if a newly implemented technology is sufficient and meets users’ intrinsically perceived requirements, it will be adopted immediately. We believe that if 5G users meet 5G’s advertised performance, they will begin to take up the habit of using 5G.

Earlier research identified health consciousness as a measure of an individual’s intrinsic motivation to preserve good health (Dutta-Bergman, 2005; Choi and Kwon, 2020). The findings of another study demonstrated three mediators in the effect of health consciousness on message acceptance (i.e., perceived intensity, response efficacy, and self-efficacy). So we can say if technology is helpful in everyday life and can minimize human effort while increasing efficiency and productivity, it would be widely adopted. The literature reviewed previously leads us to hypothesize that:

H1(a): Intrinsic factors are positively associated with 5G technology adoption.

H2: Intrinsic factors are positively associated with (a) Health consciousness attitude, (b) behavioral intention.

### Psychological Factors (Satisfaction, Habit, Hedonic Motivation, Curiosity), Health Consciousness, Behavioral Intention, and 5G Adoption

The fundamental principle of the human psyche is that if anything satisfies a human, they will use and adapt it forever. Psychology is a discipline that has wide importance in every field. Psychological factors play an important role in the decision-making of human beings. Numerous psychological factors have been studied previously, such as satisfaction, habit, hedonic motivation, and curiosity behind adopting or rejecting technology. Motivational theories stress the importance of consumer retention and consistent involvement. It was discovered that happy patients are more receptive to adopting health information technology (Roham et al., 2012). Satisfied farmers are persuasive in adopting water-saving technologies (Rouzaneh et al., 2021). According to a study conducted in China and Korea on early adopters of electric vehicles, psychological and behavioral factors positively affect consumers’ adoption (Chu et al., 2019). Habit is defined as “the extent to which people tend to perform behavior automatically because of learning” (Venkatesh et al., 2012). Browsing the internet on a smartphone *via* various applications is similar, resulting in strong usage patterns attributed to a technology habit and demonstrating technology’s role in fostering behavioral intent (Chipeva et al., 2018). Hedonic motivation is a concept that refers to the pleasure or gratification derived by an individual through the use of novel technology (Venkatesh et al., 2012). If you recognize enjoyment in technology, you will begin to accept it and more likely use it because you enjoy it (Baabdullah, 2018). Numerous recent research indicates that technology’s hedonic enjoyment enhances a person’s ability to accept and use a service (Alalwan et al., 2018; Aswani et al., 2018). Curiosity is well-defined as “a desire for information in the absence of extrinsic reward” (*International Handbook of Emotions in Education, 2014*). Existing research indicates that sensitivity to Curiosity liberation triggers striatal regions previously associated with reward-based decision making (Jepma et al., 2012). According to drive theory and incongruity theory, the need to explore and learn could be linked to the primal drives of hunger and thirst. Hunger induces eating while curiosity induces information-seeking. Current research indicates that curiosity influences behavior and that being unaware of certain factors contributes significantly to eliciting interest.

Espinosa et al. established two psychological principles as significant predictors of health behaviors: emotional intelligence and health consciousness (Espinosa and Kadic-Maglajlic, 2018). Consumers’ health consciousness and social power significantly affect their perceived value and attitude toward the brand. In addition, the perceived importance, brand attitude, and brand loyalty directly affect purchasing intent (Jinying, 2019). Ziguras, in his book Self-care: Embodiment, personal autonomy and the shaping of health consciousness, explain that psychological, social, and cultural factors shape individuals’ behavior toward health and self-care (Ziguras, 2004). Ziguras said, “You are your savior and your own worst enemy.” With this literature, we hypotheses that:

H1(b): Psychological factors are positively associated with 5G technology adoption.

H3: Psychological factors are associated with (a) Health consciousness attitude, (b) behavioral intention.

### Social Factors (Social Influence, Environmental Knowledge, and Environmental Awareness) Health Consciousness, Behavioral Intention, and 5G Adoption

Numerous technology adoption models study social factors as a predictor of technology adoption. The most significant social factors in technology adoption models are social influence, environmental knowledge, and environmental awareness. The term “social influence” refers to the extent to which an individual treats the opinions of others as important enough to justify the adoption of a revolutionary scheme (Venkatesh et al., 2003). Conformity or social influence (SI) often refers to the pressure placed on an individual’s values by others (Khan and Qudrat-Ullah, 2021). Scholars have shown that social influence significantly affects an individual’s decision to use technology-related services (Li and Zhao, 2021).

Respect for the environment mentality encompasses attitudes, concerns, and actions toward the environment and strategies and suggestions for resolving environmental problems (Wang et al., 2016; Awan et al., 2021). As a result, environmental consciousness is a necessary component of individuals’ transition from their current behaviors to more sustainable ones (S. Wang et al., 2016). Furthermore, environmental consciousness, especially in 5G technology, is a significant factor in 5G adoption (Shah et al., 2021).

Environmental knowledge entails an awareness of environmental issues and their consequences, recognition, and collective responsibility (Mostafa, 2007). Environmental intelligence alters consumer perceptions of the environment, altering consumer behavioral intentions. Individuals enlightened about a particular issue and address it more effectively are more engaged than those in the dark (Bamberg, 2003). Furthermore, it has been shown in several other research that environmental knowledge and preferences influence customers’ buying decisions. Shah et al. (2021) found that environmental awareness and environmental knowledge are positively associated with health consciousness. Hence, we hypotheses that:

H1 (c): Social factors positively influence 5G technology adoption.

H4: Social factors are positively associated with (a) Health consciousness attitude, (b) behavioral intention.

### Economic Factor (Cost Value, Facilitating Conditions), Health Consciousness, Behavioral Intention, and 5G Adoption

Economic factors play a vital role in every field of study. Economic factors play a keen role in technology adoption or rejection. People tend to adopt or reject technology in their budget or out of it. Cost value and facilitating conditions come under economic factors if we talk exclusively about technology adoption. The concept of good value can be described as having both low costs and a possible return on investment. The consumer determines which application provides a perceived benefit vs. the cost associated with that particularity, denoted by the term “the Cost value” (Venkatesh et al., 2012). According to the reasonable choice theorem, price value strikes a balance between operating expenses and potential gains (Boudon, 2008). When users of a particular technology weigh the benefits and costs, their choice appears to swing between what was offered and taken away (Baabdullah, 2018). Price was the most significant factor in consumers’ willingness to adopt emerging technologies (Dhiman et al., 2019; Gul et al., 2021).

Venkatesh et al. (2003) describe facilitating conditions “the degree to which an individual believes that an organizational and technical infrastructure exists to support the use of the system.” The researchers established that assisting factors affect an individual’s intention to use technology-related services (Li and Zhao, 2021). Facilitating conditions significantly affect technology adoption (Khan and Qudrat-Ullah, 2021). Facilitating condition is also a significant predictor in mHealth adoption during COVID 19 (Alam et al., 2021). The Sales Promotion factors have a clear and beneficial effect on health consciousness in relation to the consumption of Fresh Fruits (Tran et al., 2020). So, we can conclude that if economic factors are favorable to users, they reflect the intention and adopt new technology. This literature inspired us to hypothesize that:

H1 (d): Economic factors are associated with 5G technology adoption.

H5: Economic factors are associated with (a) Health consciousness attitude, (b) behavioral intention.

### Behavioral Intention and 5G Adoption

The model used in this study explores various factors affecting the adoption of 5G technology and previously used models and their underlying assumptions. However, it is essential to understand the users’ intentions and behaviors about technology adoption. The most significant determinant is the behavioral intention behind actual user behavior (Wang et al., 2018). A fundamental tenet of adoption is that an individual’s “intention” to incorporate new technology serves to predict its “actual use” (Davis and Davis, 1989; Ajzen, 1991; Khan and Qudrat-Ullah, 2021). Numerous researchers used the variables “intention to use” and “real use” to describe technological acceptance in their research (Davis and Davis, 1989; Venkatesh et al., 2012; Alam et al., 2021). As described variables, the researchers used actual behaviors and behavioral intent interchangeably. According to previous research, individuals who believe they can use emerging technologies are more likely to do so (Ajzen, 1991). Venkatesh et al. (2003) found that “on the other hand, behavioral intention” and “actual use” are equally crucial for technology acceptance. These two critical criteria are used to assess a technology’s acceptability in the business world of literature (Alam et al., 2021). It is demonstrated that an individual’s reaction to technology use is directly related to their acceptance. Thus, behavioral intention impacts “adoption behavior” (Ajzen, 1991; Venkatesh et al., 2003, 2012). According to the existing literature, behavioral intention (BI) uses as a predictor of 5G technology adoption in this study.

H7: Behavioral intention is positively associated with 5G technology adoption.

### Health Consciousness Attitude, Behavioral Intention, and Adoption

Attitude is a term that refers to a person’s attitude toward someone or something (ElHaffar et al., 2020). It exerts a significant impact on our choices and behavior. An attitude is determined by education, climate, and experience. It is to the degree to which an individual is aware of their well-being and how that one’s health problems are dealt with (Wang et al., 2019). Previously conducted research identified health consciousness as a proxy for an individual’s intrinsic motivation to maintain good health (Dutta-Bergman, 2005; Choi and Kwon, 2020). Another study found that health consciousness and purchase decision are positively associated (Nagaraj, 2021). A study conducted in China to check the green furniture purchase attention found that health consciousness is positively associated with purchase intention (Xu et al., 2020). People who are health conscious are more mindful of and worried about their health. They choose healthy environments and goods (Lee and Sozen, 2020). As a result, individuals with a firm grasp of environmental and health concerns are more pleased with environmentally friendly goods and technologies (Shah et al., 2021). Specifically, about cellular technologies, people have mix opinion some treat it as green innovation while others talk about radiation generate to stimulate the signals. Based on these findings of previous studies we hypothesize that:

H6: Health consciousness attitude is positively associated with 5G technology adoption.

H8: Health consciousness attitude is positively associated with behavioral intention.

### The Mediating Role of Health Consciousness Attitude

Environmental awareness and environmental knowledge are significantly associated with health consciousness, while health consciousness attitude is positively associated with 5G adoption intention (Shah et al., 2021). A study on Chinese urban residents in 2020 revealed that health consciousness mediates the relationship between suboptimal health status and lifestyle (Xue et al., 2020). In another study, researchers found that the impact of conservation and self-transcendence mediates thorough health consciousness on health message credibility, health message attention, and behavioral intention (Chang, 2020). They also believe that the credibility of health messages is a psychological construct, attention to them is a cognitive construct, and compliance is a behavioral construct. As a result, causality can flow from psychological and cognitive to behavioral strategies. Health consciousness also plays a mediating role between the gender of consumers and the intention to use a menu label (Lee and Sozen, 2020). A study measuring health-related behaviors among adults revealed that health consciousness is a statistically significant mediator between health behavior and emotional intelligence (Espinosa and Kadic-Maglajlic, 2018). After analyzing this literature, we believe that health consciousness attitude mediates the relationship of our predictors and 5G technology adoption. Hence, we hypothesize that:

H9: Health consciousness attitude mediates the relationship between Intrinsic (a), psychological (b), social (c), and economic (d) factors and 5G technology adoption.

### The Mediating Role of Behavioral Intention

Behavioral intention’s mediating role is widely studied in various aspects. It is a significant mediator between adoption/usage behavior and different predictors. For example, BI plays a mediating role in adopting mobile learning between performance expectancy, social influence, effort expectancy, and user behavior (Chao, 2019). “Consumer trust and purchase intention play a multi-step mediating role in the relationship between corporate social responsibility image of the organic food company and customer co-developing behavior” (Yu et al., 2021). Purchase behavioral intention also plays a significant mediating role between online shopping behavior, perceived usefulness, and subjective norm (Lim et al., 2016). We also believe that:

H10: Behavioral intention mediates the relationship between Intrinsic (a), psychological (b), social (c), and economic (d) factors and 5G technology adoption.

## Methodology

### Research Context

In light of our study’s objective, we chose to test our model in China. The explanation for this choice is that China was one of the first countries to deploy 5G technology and is the world’s most populated country, making it the largest market for any technology. To ensure that our study’s findings are consistent and accurate, we gathered responses from Chinese citizens and foreigners living in China. In addition, we distributed our questionnaire in all of the country’s major cities to secure the most accurate data.

### Construction of Instrument and Collection of Data

We used a validated construct from previous research. Each measurement object’s layout and specifications are detailed in Supplementary Appendix 1. Before a formal survey, we perform a pilot study to ascertain respondents’ consumption time and feedback. Twenty master’s and doctoral students were chosen for a pilot project. At the next step, we work with two Chinese-speaking translators. Language experts translate the instrument’s English edition into Chinese to meet the specific requirement of our respondents. Then, we retranslate the instrument into English to check whether it accurately calculates the same response as the instrument’s original version. Academic experts were consulted for the translated Chinese version of the instrument to ensure that build objects had the same significance in Chinese and English. Finally, fifteen Chinese master’s level students were chosen for the final instrument test. They provided positive reviews, and the findings of the second phase pilot study encouraged the continuation of the study – pilot study participants were not included in the final sample.

We conducted an online survey to prevent manual data entry and other human errors. We contacted several expatriate online community groups to elicit responses from international residents in China. Additionally, the questionnaire has been shared with Chinese 5G users in major cities of China to create a more diverse sample for a more thorough analysis. Participants for this study were picked using a non-probability sampling technique. It is a fair method of presenting a comprehensive view of the phenomenon (Sekaran and Bougie, 2010).

It took 9 weeks to collect 830 credible responses (Table 1). All variables were quantified using a seven-point Likert scale, with 1 denoting “strongly disagree” and 7 denoting “strongly agree.” Seven-point Likert items are more concise, easier to administer, and reflect a respondent’s accurate evaluation more accurately. As a result of these advantages, 7-point survey questions are often preferred over higher-order alternatives (Finstad, 2009).

**TABLE 1:** Demographic profile of the respondents.

		Frequency	Percent
Gender	Male	446	53.70%
	Female	384	46.30%
	Total	830	100.00%
Age	18–25 Year	202	24.30%
	26–35 Year	272	32.80%
	36–45 Year	275	33.10%
	>45 Year	81	9.80%
	Total	830	100.00%
Education	High School	135	16.30%
	Bachelor	322	38.80%
	Master	347	41.80%
	Doctorate	26	3.10%
	Total	830	100.00%
Occupation	Student	216	26.00%
	Govt. Employe	170	20.50%
	Private Company Employe	263	31.70%
	Businessman/women	181	21.80%
	Total	830	100.00%
Tenure Using 5G	<3 Months	165	19.87%
	3–6 Months	317	38.10%
	>6 Months	348	41.92%
	Total	830	100.00%
Residential Status	Chinese Citizen	502	60.50%
	Expatriate	328	39.50%
	Total	830	100.00%

## Data Analysis

### Structural Equation Modeling vs. Fuzzy Set Qualitative Comparative Analysis

This study investigates the relationship between the key factors of 5G technology adoption using both the SEM and the *fs*QCA. These two approaches are distinct in their objectives and predicated on distinct ideas. The SEM is a variable-oriented technique that emphasizes the independent variable’s net effect on the dependent variable; it views independent variables as competitors for explaining variation in the dependent variables. It is based on additive effects and linearity (Woodside, 2013). In comparison, the *fs*QCA technique is a case-based approach that focuses on combinatorial effects. In this model, the key assumption is that numerous possible solutions or pathways lead to the same outcome. The same factors can lead to multiple outcomes in this strategy, while distinct conditions may be essential or sufficient to cause a certain outcome (Rihoux and Ragin, 2008; Woodside, 2013).

#### Partial Least Squares-Structural Equation Modeling

Given that intrinsic, psychological, social, and economic factors are a second-order formatively measured Construct, “the Partial Least Squares Structural Equation Modeling (PLS-SEM), a type of composite-based SEM, is the optimal first stage of analysis in this study when compared to factor-based SEM such as AMOS and LISREL” (Woodside, 2013). We employed PLS-SEM because “this technique is most recommended when the research focuses on predicting and exploring the dependent variables to explain the maximum variance. So, PLS-SEM is the best prediction-oriented approach” (Hair et al., 2016). “It can also handle the measurement (outer) and structural (inner) models simultaneously. Further, it is appropriate for analyzing complex path models” (Hair et al., 2016). Finally, “the PLS-SEM can also cater to a small sample size and provide more accurate results.” Thus, PLS-SEM appears to be a suitable method for our research. The usage of the PLS-SEM methodology is on the increase, as recently pointed out, owing to its possible benefits in Management science (Hair et al., 2016).

The PLS path modeling method is assessed in two steps to ensure that the constructs’ measures are accurate and reliable: (a) Measurement model assessment indicates reliability and Validity of the outer mode, and (b) the structural model assessment defines the inner model or relationship among the latent constructs. Nonetheless, because the model contains non-linear relationships, it is advantageous to undertake a dual-stage analysis.

#### Fuzzy Set Qualitative Comparative Analysis

Configurational techniques have gained popularity over several years, with *fs*QCA playing a significant role. The *fs*QCA has been used in knowledge management (Sahibzada et al., 2020), organizational performance (Farooq Sahibzada et al., 2021), consumer psychology (Schmitt et al., 2017), online business and marketing (Pappas, 2018), information systems (Liu et al., 2017), strategy, and organizational research (Fiss, 2011).

Fuzzy set qualitative comparative analysis is advantageous for inductive and deductive reasoning in developing, elaborating, and testing theories (Pappas and Woodside, 2021). Researchers can conduct extensive research on various explanations for the outcome of interest or concentrate on testing a select set of hypotheses (Pappas, 2018) based on theory or past findings. Locating, identifying, and verifying specific examples in the sample; locating and investigating other possible models to explain high scores for the same outcome; *fs*QCA is helpful (Pappas and Woodside, 2021). The researcher can use the additional contextual information and findings discovered to explain and discuss the findings. Variance-based analysis typically identifies a single optimal solution, restricting the results (Woodside, 2013). Combining *fs*QCA with additional data analysis techniques should yield improved results (Schneider and Wagemann, 2010).

With the implementation of *fs*QCA, it is possible to obtain various benefits compared to traditional analytical methods. Capturing the combinations of conditions necessary for an outcome to occur, *fs*QCA combines qualitative and quantitative assessments and computes the degree to which a case belongs to a set (Rihoux and Ragin, 2008), bridging the qualitative and quantitative domains. The primary advantages of *fs*QCA compared to conventional variance-based approaches and their inherent constraints (Woodside, 2013; Liu et al., 2017). Variance-based methods investigate variables in a competing context as they compute the net influence between variables in a model and tend to utilize less rigorous methodologies and simpler models. At the same time, FSQCA goes deeper and develops more complicated and asymmetric models (Pappas and Woodside, 2021). FsQCA uses combinations of independent variables that include not identified using traditional variance-based approaches because they capture just the major effects (Woodside, 2014). FsQCA divides the sample into various subsets, examining multiple conditions in combination. An outlier can be present in some but not all feasible solutions (Pappas and Woodside, 2021). In this sense, *fs*QCA has a sort of increased robustness over variance-based (v-b) approaches, as the v-b approach is not susceptible to outliers (Fiss, 2011; Liu et al., 2017).

### Multivariate Assumptions

According to Hair et al. (2016), the multivariate assumptions of normality, linearity, multicollinearity, and homoscedasticity must be examined before doing any multivariate tests. We protected respondents’ privacy throughout data gathering and explained that there is no right or wrong answer. The Kolmogorov-Smirnov (K-S) test was used to determine the normality of the data distribution, but the results indicate that the data are not normal. Finally, the variance inflation factor (VIF) scores were analyzed to evaluate whether the model had collinearity difficulties. For Hair et al. (2014), VIF values under 5 signify that the acquired data do not exhibit collinearity concerns. The results of this study indicate that all variables have VIF values that are lower than 5.

Given that the VIF values ranged between 1.225 and 2.908, it is determined that there is no multicollinearity among the first-order constructs Table 2. Thus, it demonstrates that the current study’s data are not collinear and reaffirms the model’s robustness. Finally, the scatter plot of the regression standardized projected value and the residual value reveals that the evidence supports this assumption.

**TABLE 2:** Reliability and validity analysis.

*Constructs*	*Items*	*Loadings*	*T-values*	*VIF*	*C*α	*CR*	*AVE*
Perceived Performance	PP1	0.877	71.960	2.079	0.851	0.910	0.771
	PP2	0.877	72.856	2.055			
	PP3	0.880	81.280	2.117			
Perceived Functional Value	PFV1	0.922	119.08	2.096	0.839	0.926	0.861
	PFV2	0.934	170.32	2.096			
Perceived Value	PV1	0.858	67.038	2.430	0.869	0.911	0.719
	PV2	0.873	65.558	2.607			
	PV3	0.862	47.495	2.381			
	PV4	0.797	36.933	1.870			
Satisfaction	SAT1	0.691	26.133	1.328	0.723	0.828	0.547
	SAT2	0.691	27.223	1.312			
	SAT3	0.768	42.867	1.547			
	SAT4	0.801	56.252	1.593			
Habit	HAB1	0.895	93.530	2.346	0.857	0.913	0.778
	HAB2	0.897	104.94	2.380			
	HAB3	0.853	64.840	1.892			
Hedonic Motivation	HM1	0.861	87.105	1.829	0.833	0.900	0.750
	HM2	0.866	70.927	2.003			
	HM3	0.870	74.119	1.972			
Curiosity	CUR1	0.746	44.724	1.558	0.820	0.882	0.651
	CUR2	0.849	80.400	2.074			
	CUR3	0.832	62.679	2.185			
	CUR4	0.797	45.850	1.938			
Social Influence	SI1	0.794	47.714	1.394	0.717	0.841	0.638
	SI2	0.808	51.676	1.435			
	SI3	0.795	50.363	1.385			
	EK1	0.872	3.0060	1.716	0.772	0.866	0.683
Environmental Knowledge	EK2	0.797	2.1620	1.714			
	EK3	0.808	2.8550	1.433			
Environmental Awareness	EA1	0.753	41.382	1.365	0.715	0.823	0.538
	EA2	0.753	41.665	1.343			
	EA3	0.751	41.208	1.361			
	EA4	0.672	26.105	1.313			
Facilitating Condition	FC1	0.877	80.653	2.609	0.909	0.936	0.786
	FC2	0.896	90.899	2.908			
	FC3	0.885	88.086	2.751			
	FC4	0.890	92.591	2.827			
Cost Value	CV1	0.840	53.720	1.764	0.839	0.903	0.756
	CV2	0.879	69.558	2.156			
	CV3	0.889	77.658	2.128			
Health Consciousness Attitude	HCA2	0.873	71.915	1.499	0.732	0.881	0.788
	HCA3	0.902	128.44	1.499			
Behavioral Intention	BI1	0.893	76.936	2.257	0.882	0.927	0.809
	BI2	0.905	103.49	2.726			
	BI3	0.900	92.405	2.544			
Technology Adoption	TA1	0.749	36.659	1.243	0.642	0.807	0.583
	TA2	0.741	35.721	1.225			
	TA3	0.800	47.995	1.344			

### Measurement Model

Hair et al. (2016) suggested that measurement model assessment is based on the reliability of the indicator and construct convergent Validity and discriminant validity. Cronbach’s Alpha (Cα) and indicator loading were employed to evaluate the instrument’s reliability. The indicator of the constructs was tested for convergent Validity to determine whether they accurately assess the study variables. The total sum of variance in the indicators compensated for by the latent Construct is represented by AVE, while CR specifies the variables’ consistency. Individual items’ reliabilities are reported in Table 2 and assessed through factor loadings of the items on the corresponding constructs. Only those items containing factor loading equal to or greater than 0.6; have been considered significant and retained in the model (Hair et al., 2016). Only four HAB4, SI4, FC5, and HCA1 items have a lower value, so we removed them from the final model. Moreover, the importance of Cronbach’s alpha of all constructs is more significant than or very close to 0.7, which is acceptable (Werts et al., 1974).

Additionally, for further strengthens the assessment of the reliability of the Construct. The composite reliability of the constructs has also been assessed because it is commonly admitted that composite reliability is a more effective tool to measure the reliability than Cronbach’s alpha (Werts et al., 1974). Composite reliability values of constructs are also greater than 0.7, which further toughens the reliability of variables. Simultaneously, the average variance extracted (AVE) estimates for all constructs were greater than 0.50, establishing the convergent Validity (Hair et al., 2016; see Table 2).

In addition, to validate the proposed model’s discriminant Validity, the Fornell-Larcker criteria are applied (Hair et al., 2016). Table 3 indicates that discriminant validity is confirmed based on the Fornell-Larcker criteria since the top value of the association of measures is maximum in each column (Fornell and Larcker, 1981; Hair et al., 2016). Furthermore, we have also applied the HTMT ratio (Table 4) criterion for discriminant validity because the researcher criticized the Fornell-Larcker criteria and argued that it could not identify the absence of discriminant validity (Henseler et al., 2015).

**TABLE 3:** Fornell-Larcker criterion.

	BI	CUR	CV	EA	EK	FC	HAB	HM	HC	PFV	PP	SAT	PV	SI	TA
BI	0.900														
CUR	0.522	0.807													
CV	0.561	0.612	0.869												
EA	0.638	0.361	0.395	0.733											
EK	0.060	0.048	0.037	0.068	0.826										
FC	0.796	0.482	0.602	0.591	0.063	0.887									
HAB	0.458	0.610	0.542	0.281	0.013	0.422	0.882								
HM	0.526	0.504	0.491	0.378	0.008	0.526	0.617	0.866							
HCA	0.705	0.440	0.388	0.550	0.119	0.601	0.420	0.485	0.888						
PFV	0.623	0.401	0.407	0.379	0.004	0.536	0.389	0.424	0.443	0.928					
PP	0.765	0.449	0.565	0.524	0.072	0.768	0.465	0.509	0.524	0.551	0.878				
SAT	0.545	0.458	0.397	0.366	0.070	0.580	0.511	0.517	0.618	0.433	0.589	0.739			
PV	0.229	0.130	0.168	−0.012	−0.018	0.198	0.143	0.117	0.194	0.270	0.183	0.141	0.848		
SI	0.469	0.590	0.533	0.311	0.002	0.430	0.564	0.423	0.325	0.353	0.472	0.428	0.110	0.799	
TA	0.590	0.408	0.332	0.466	0.059	0.504	0.284	0.346	0.484	0.322	0.429	0.424	0.095	0.341	0.764

**TABLE 4:** Heterotrait-Monotrait ratio (HTMT).

	BI	CUR	CV	EA	EK	FC	HAB	HM	HC	PFV	PP	SAT	PV	SI	TA
BI															
CUR	0.612														
CV	0.647	0.732													
EA	0.816	0.474	0.509												
EK	0.066	0.064	0.056	0.087											
FC	0.787	0.557	0.686	0.740	0.074										
HAB	0.526	0.727	0.633	0.365	0.025	0.478									
HM	0.614	0.606	0.581	0.497	0.042	0.605	0.728								
HCA	0.871	0.567	0.494	0.762	0.152	0.738	0.532	0.622							
PFV	0.722	0.482	0.483	0.497	0.024	0.611	0.460	0.506	0.567						
PP	0.783	0.536	0.666	0.690	0.087	0.772	0.545	0.604	0.665	0.651					
SAT	0.678	0.587	0.497	0.522	0.092	0.713	0.649	0.658	0.749	0.555	0.750				
PV	0.259	0.154	0.196	0.080	0.067	0.222	0.167	0.137	0.243	0.314	0.213	0.179			
SI	0.589	0.769	0.686	0.438	0.038	0.532	0.719	0.545	0.446	0.456	0.605	0.593	0.141		
TA	0.782	0.561	0.450	0.713	0.089	0.660	0.381	0.474	0.698	0.436	0.580	0.616	0.126	0.502	

*Gray boxes serve as a separator between HTMT ratio values and blank cells.*

### Structural Model Assessment

The second phase in PLS-SEM evaluation is structural model assessment. The assessment of the structural path model involves assessing the multicollinearity, the model’s predictive relevance and empirical significance of path coefficients, and the confidence level (Hair et al., 2020). The present study employed general recommendations to evaluate the structural model and interpret findings (Hair et al., 2014). However, specific recommendations from Hair et al. (2020) were also considered for mediation analysis.

**FIGURE 2:**
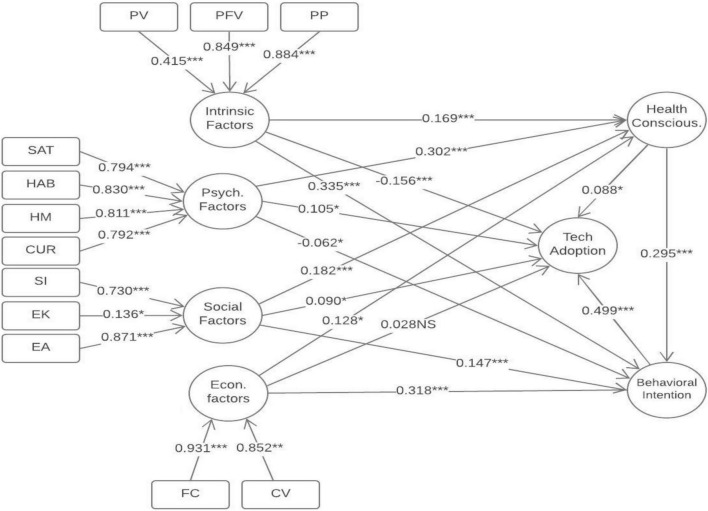
Path model.* significant level 0.001; ** significant level <0.01; *** significant level <0.05; NS=not supported.

Furthermore, we have three endogenous constructs in our model (see Figure 2). The R^2^ for technology adoption was 0.377 (*Q*^2^ = 0.216), Health consciousness attitude (HCA) was 0.449 (*Q*^2^ = 0.351), and behavioral intention (BI) was 0.773 (*Q*^2^ = 0.619) which indicates that 37.7, 44.9, and 77.3% of the variance in the respective constructs can be explained by their predictors. Q^2^ values greater than 0 indicate sufficient predictive relevance (Table 4). Furthermore, the effect size (*f*^2^) results are presented in Table 5. The finding reveals that the values for *f*^2^ of the relationships are lower to higher levels, confirming model fitness (Meng and Bari, 2019).

**TABLE 5:** Variance inflation factor, coefficient of determination and predictive relevance.

	*VIF*			*R* ^2^	*Q* ^2^
Constructs	Behavioral Intention	Health Conscious	Technology Adoption		
Behavioral Intention			4.408	0.773	0.619
Economic Factors	3.093	3.063	3.54		
Health Conscious	1.814		2.197	0.449	0.351
Intrinsic Factors	2.333	2.281	2.828		
Psychological Factors	2.392	2.226	2.408		
Social Factor	2.001	1.941	2.096		
Technology Adoption				0.377	0.216

Next, we ran a bootstrapping of 5,000 resamples to assess the developed hypothesis. First, we assessed the direct relationships before looking at the mediation effects. Table 5 and Figure 2 indicate all the direct relationship results. Moreover, for H1 to H1d findings show that Intrinsic Factors (β = −0.156; *p* < 0.001), Psychological Factors (β = 0.105; *p* = 0.023), Social Factor (β = 0.090; *p* = 0.028) are significantly associated with Technology Adoption, but Economic factors (β = 0.028; *p* = 0.514) are insignificantly associated with Technology Adoption, which supports H1a, H1b, and H1c, while H1d is not supported. For H2a and H2b, findings reveal that Intrinsic factors are significantly associated with HCA (β = 0.169; *p* < 0.001) and BI (β = 0.335; *p* < 0.001). Hence H2a and H2b are supported. In addition, For H3a and H3b, findings reveal that Psychological Factors are positively associated with HCA (β = 0.302; *p* < 0.001) and negatively associated with BI (β = −0.062; *p* < 0.018). Hence H3a and H3b are also give support for the current study. Moreover, For H4a and H4b, findings reveal that social factors are significantly associated with HCA (β = 0.182; *p* < 0.001) and BI (β = 0.147; *p* < 0.001). Hence H4a and H4b are supported. Similarly, For H5a and H5b, the results show that EF are also positively associated with HCA (β = 0.128; *p* = 0.011) and BI (β = 0.318; *p* < 0.001). Hence H5a and H5b are also supported. For H6 and H7, findings indicate that HCA (β = 0.088; *p* < 0.024) and BI (β = 0.499; *p* < 0.001) are significantly and positively associated with Technology adoption, which gives support for H6 and H7. The study proposed the relationship between HCA and BI in the last direct relationship, and findings revealed the significant relationship between the latent constructs (β = 0.295; *p* < 0.001). Hence H8 was supported for the current study.

To examine the mediating effects of HCA and BI, we performed further analyses to verify the indirect effects’ magnitude and statistical significance. As (Preacher and Hayes, 2008) suggested, this study used the bootstrap method with 5,000 iterations to analyze the significance of indirect effects (Table 5).

Additionally, the hypothesis tests were conducted after analyzing age, education, and gender control roles, and Table 5 shows that control variables had no significant effect on technology adoption.

Table 6 demonstrates that the indirect effects of Intrinsic factors (β = 0.015; *p* = 0.039), Psychological factors (β = 0.027; *p* = 0.036), and social factors (β = 0.016; *p* = 0.043) through HCA on Technology adoption are statistically significant, while the indirect effects of Economics factors through health conscious on Technology adoption are not supported (β = 0.011; *p* = 0.122). Therefore, hypothesis H9a, H9b, and H9c are supported, while H9d is not supported. Moreover, the indirect effects of Intrinsic factors (β = 0.167; *p* < 0.001), Psychological factors (β = −0.031; *p* = 0.026), social factors (β = 0.073; *p* < 0.001) and Economics factors (β = 0.159; *p* < 0.001) through BI on Technology adoption are supported. Hence, hypothesis H10a, H10b, H10c and H10d are supported.

**TABLE 6:** Direct effects.

*Hypotheses*	*Relationships*	*Beta*	*SD. dev*	*T-Values*	*P-Values*	*2.5%*	*97.5%*	*F* ^2^	*Supported*
H1a	Intrinsic Factors -> Technology Adoption	−0.156	0.043	3.649	0.000	−0.241	−0.075	0.014	Yes
H1b	Psychological Factors -> Technology Adoption	0.105	0.046	2.268	0.023	0.014	0.193	0.007	Yes
H1c	Social Factor -> Technology Adoption	0.090	0.041	2.202	0.028	0.011	0.170	0.006	Yes
H1d	Economic Factors -> Technology Adoption	0.028	0.043	0.653	0.514	−0.056	0.117	0.000	No
H2a	Intrinsic Factors -> Health Conscious	0.169	0.040	4.192	0.000	0.090	0.245	0.023	Yes
H2b	Intrinsic Factors -> Behavioral Intention	0.335	0.030	11.11	0.000	0.278	0.395	0.212	Yes
H3a	Psychological Factors -> Health Conscious	0.302	0.046	6.578	0.000	0.210	0.392	0.074	Yes
H3b	Psychological Factors -> Behavioral Intention	−0.062	0.026	2.366	0.018	−0.113	−0.011	0.007	Yes
H4a	Social Factor -> Health Conscious	0.182	0.043	4.207	0.000	0.098	0.268	0.031	Yes
H4b	Social Factor -> Behavioral Intention	0.147	0.026	5.713	0.000	0.096	0.197	0.047	Yes
H5a	Economic Factors -> Health Conscious	0.128	0.050	2.542	0.011	0.028	0.229	0.010	Yes
H5b	Economic Factors -> Behavioral Intention	0.318	0.032	9.919	0.000	0.255	0.380	0.144	Yes
H6	Health Conscious -> Technology Adoption	0.088	0.039	2.254	0.024	0.009	0.165	0.006	Yes
H7	Behavioral Intention -> Technology Adoption	0.499	0.048	10.29	0.000	0.405	0.596	0.091	Yes
H8	Health Conscious -> Behavioral Intention	0.295	0.023	12.72	0.000	0.248	0.340	0.211	Yes
*Control Variables*		*Beta*	*SD. dev*	*T-Values*	*P-Values*	*2.5%*	*97.5%*	−	*Supported*
Age -> Technology Adoption		0.003	0.035	0.094	0.925	−0.065	0.072	−	No
Education -> Technology Adoption		−0.040	0.035	−1.160	0.246	−0.109	0.028	−	No
Gender -> Technology Adoption		−0.009	0.035	−0.252	0.801	−0.077	0.060	−	No

### Calibration

Because the *fs*QCA algorithm makes use of the concept of set membership, the raw data must be translated into fuzzy sets ranging from zero (complete omission from a set) to one (full inclusion) (Ragin, 2009). Before calibrating the variables, the study creates an index for each Construct by averaging the related indications. Three anchors are required during the calibration process: complete membership, full non-membership, and a cross-over point (Ragin, 2009). The study calibrates the fuzzy sets based on the theoretical anchors (Frösén et al., 2016; Silva and Gonçalves, 2016; Pappas and Woodside, 2021) using the direct method for all constructs (Ragin, 2009). In this study, we have used seven-point Likert scales so, we have followed the instructions of Pappas and Woodside (2021) and used the threshold of 6 for full set membership, 4 as cross-over point, and 2 as full set non-membership. We have used *fs*QCA software version 3.1 for data calibration in our study. Cases exactly on 0.5 are dropped from the analysis in *fs*QCA; it becomes difficult to analyze the exact conditions on 0.5 (i.e., intermediate-set membership) (Ragin, 2009). Fiss (2011) proposes resolving this issue by introducing a constant of 0.001 to the causal conditions below complete membership scores of 1. We have followed (Fiss, 2011) advice and added 0.001 constant to avoid this issue.

### Contrarian Case Analysis

We have followed (Pappas and Woodside, 2021) and performed contrarian case analysis before applying *fs*QCA. It enables an easy and rapid examination of the proportion of instances in our sample that is not explained by main effects and hence would be excluded from the outcome of a normal variance-based method (Woodside, 2014, 2016). Contrarian cases do arise independently of the significance of the major impacts; hence the presence of these cases can be determined by a contrarian case study (Woodside, 2014).

We divide the sample into quintiles. We avoid doing median splits on continuous variables to minimize a reduction in statistical power and the likelihood of misleading results (Fitzsimons, 2008). Next, we did cross-tabulations to calculate the degrees of connection between the quintiles, which cross-tabs allow us to compute. The results are shown in Supplementary Appendix 2. The primary effects are presented in the top left and bottom right cases, while the non-main effects are shown in the bottom left and top right situations. These are situations that are contrary to the majority in our dataset. The findings indicate several interactions between variables independent of the primary effect, indicating the importance of performing a configurational analysis.

### Analysis of Necessary Conditions

We first investigate if any of the causative conditions are necessary before advancing to the next stage of *fs*QCA (i.e., generating a truth table). The results of the *fs*QCA study are interpreted based on consistency. The condition is necessary when the consistency is above 0.9 (Schneider and Wagemann, 2012). Using an SEM model, this study evaluates one endogenous variable, 5G technology adoption (Figure 1). Like SEM, *fs*QCA also considers six antecedent conditions for the outcome (5G technology adoption). The findings of the *fs*QCA analysis on the necessity of the conditions necessary for 5G technology adoption are presented in Table 7. Results revealed that the consistency of “bi” is above 0.90, so it will be necessary for 5G technology adoption.

**TABLE 7:** Meditation results.

*Hypotheses*	Relationships	Beta	SD. dev	*T*-values	*P*-Values
H9a	Intrinsic Factors -> Health Conscious -> Technology Adoption	0.015	0.008	1.973	0.039
H9b	Psychological Factors -> Health Conscious -> Technology Adoption	0.027	0.013	2.101	0.036
H9c	Social Factor -> Health Conscious -> Technology Adoption	0.016	0.008	2.027	0.043
H9d	Economic Factors -> Health Conscious -> Technology Adoption	0.011	0.007	1.545	0.122
H10a	Intrinsic Factors -> Behavioral Intention -> Technology Adoption	0.167	0.023	7.243	0.000
H10b	Psychological Factors -> Behavioral Intention -> Technology Adoption	−0.031	0.014	2.219	0.026
H10c	Social Factor -> Behavioral Intention -> Technology Adoption	0.073	0.014	5.306	0.000
H10d	Economic Factors -> Behavioral Intention -> Technology Adoption	0.159	0.023	6.907	0.000

### Obtaining the Fuzzy Set Qualitative Comparative Analysis Solutions

Once the calibration and necessary conditions analysis is complete, the “*fs*QCA algorithm generates a truth table of 2*^k^* rows, where “k” represents the number of outcome predictors. Each row represents each potential combination. The truth table should be fine-tuned in frequency and consistency” (Ragin, 2009). The “frequency refers to the total number of observations made for each feasible combination.” Consistency refers to *“the degree to which cases correspond to the set-theoretic relationships expressed in a solution”* (Fiss, 2011). A frequency cut-off point is established to guarantee adequate empirical observations for assessing subgroup relationships. A cut-off point of 1 is adequate for small and medium-sized samples, but for large-scale samples (e.g., 150 or more cases), the cut-off value should be set higher (Ragin, 2009). Following the previous researchers (Fiss, 2011; Pappas and Woodside, 2021), we have set a frequency cut-off point at 4, and the level of consistency permissible for observations is at 0.75 (Ragin, 2006). The remaining configurations accounted for more than 80% of the cases, according to Ragin (2009) advice. After applying these cut-off points, the Quine-McCluskey algorithm yielded three solutions: a complex solution, a parsimonious solution, and an intermediate solution. We have followed (Fiss, 2011) in results interpretation as he has stated that, by combining the parsimonious and intermediate solutions, a more thorough and aggregated perspective of the findings can be obtained (Fiss, 2011).

Additionally, these solutions are supported by essential and sufficient conditions classified as core or peripheral. “Core” conditions or elements, i.e., essential elements, cannot be omitted from any configuration (i.e., elements that have a strong causal relationship with the outcome) and are frequently seen in the parsimonious and intermediate solutions. On the other hand, “peripheral” conditions or parts are less critical and perhaps interchangeable (i.e., elements having a weak causal relationship to the outcome), and they typically exist only in the intermediate solution (Kourouthanassis et al., 2017). Additionally, these requirements may be present or absent in a solution, or they may be irrelevant (i.e., “do not care”) (Fiss, 2011). Conditions may be present or absent in a “do not care” situation.

To aid in understanding the results, we identified and reported the core and peripheral conditions in each configuration, as suggested by Pappas and Woodside (2021). To illustrate, the black circles (•) and crossed-out circles (⊗) denote the presence and absence, respectively. Large circles denote core conditions, small circles suggest periphery conditions, and blank spaces denote a state of “do not care.” As presented in Table 8, the overall consistency of the solution was 0.76, showing that the causal combination ensures the outcome (i.e., 5G Technology adoption). The results indicated that the total solution achieved a high degree of coverage (0.829), indicating that 82.9% of cases with a high level to adopt 5G technology exhibited these six causal conditions. Similarly, all detected configurations demonstrated good consistency (above the threshold level of 0.75) and coverage, indicating that the model was informative (Woodside, 2013). The overall solution coverage indicates the extent to which 5G technology adoption can be predicted from the collection of configurations and is comparable to the R-square value stated in correlational approaches.

**TABLE 8:** Necessity analysis.

*Condition tested*	*Consistency*	*Coverage*
bi	***0.916***	*0.708*
∼bi	*0.236*	*0.465*
pf	*0.877*	*0.703*
∼pf	*0.285*	*0.515*
Sf	*0.898*	*0.673*
∼Sf	*0.282*	*0.604*
*ef*	*0.880*	*0.706*
∼*ef*	*0.290*	*0.524*
*hca*	*0.880*	*0.708*
∼*hca*	*0.251*	*0.450*
*if*	*0.770*	*0.626*
∼*if*	*0.412*	*0.722*

*The symbol ∼ denotes the absence of the condition.*

### Predictive Validity

**FIGURE 3:**
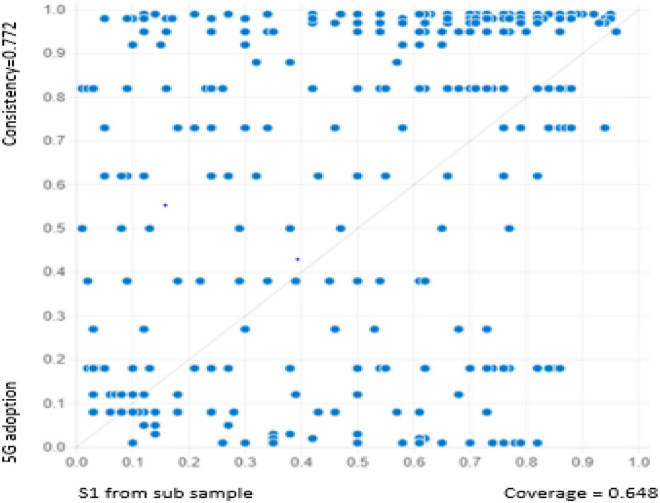
Fuzzy-plot of solution 1 (Table 10) using holdout dataset.

It is critical to validate our solutions (models) for predictive Validity. The predictive Validity is determined by how well it predicts the dependent variable in different samples (Gigerenzer and Brighton, 2009; Woodside, 2014). Predictive Validity is crucial since a good model fit does not necessarily imply that the model makes accurate predictions in all situations. For this purpose, we followed the instruction of earlier researchers (Pappas and Woodside, 2021) and split our sample into holdout samples and subsamples (415 observations for each). We run the same analysis and select the same cut-off points applied at the full sample. The set of solutions achieved from the subsample is presented in Table 9. In the second step, we computed a new solution 1 using the holdout dataset. We then plot the “XY Plot” (Figure 3) and compare the results of solution 1 and “XY Plot.” Solution 1 provided us a raw coverage of 0.645 with a consistency of 0.779, while “XY Plot” reflects the same results, i.e., coverage of 0.648 with the consistency of 0.772 for 5G technology adoption. These calculations indicate that data are largely consistent (77%), claiming that solution 1 is a subset of 5G technology adoption and its coverage is (78%). All models, predictive tests have revealed that the highly consistent models for the subsample have strong predicting abilities for the holdout sample and vice versa. On request, readers will be provided with all of the results.

**TABLE 9:** *fs*QCA findings.

	Solutions for 5G technology adoption					
Configurations	1	2	3	4	5	6
IF		•	⊗	•	•	•
PF	●	●				⊗
SF	•	•	•	•	⊗	•
EF			•	•	•	●
HCA	•		•	⊗	●	●
BI	●	●	●	●	•	⊗
Consistency	*0.785*	*0.775*	*0.821*	*0.762*	*0.765*	*0.787*
Raw coverage	*0.740*	*0.647*	*0.330*	*0.180*	*0.191*	*0.117*
Unique Coverage	*0.076*	*0.008*	*0.002*	*0.014*	*0.006*	*0.0142*
*Overall solution consistency*	*0.763*					
*Overall solution coverage*	*0.829*					

*Black circles (•) indicates the presence of a condition, and circle with “×” (⊗) indicate its absence, while blank space indicates “don’t care condition.”*

*Large circle: core condition, small circle: Peripheral condition.*

*IF, intrinsic factors; PF, psychological factors; SF, social factors; EF, economic factors; HCA, health consciousness attitude; BI, behavioral intention.*

**TABLE 10:** Solutions (models) from the subsample.

	*Models from subsamples*	*Raw coverage*	*Unique coverage*	*consistency*
S1	if2*sf2*pf2*bi2	0.645	0.060	0.779
S2	if2*hca2*ef2*∼sf2*bi2	0.201	0.014	0.770
*S3*	hca2*ef2*sf2*pf2*bi2	0.704	0.120	0.790
*Overall solution coverage: 0.779*				
*Overall solution consistency: 0.769*				

## Discussion

This study identifies an empirically tested health consciousness-based integrated model of UTAUT2 for technology adoption. We have tested our model on 5G technology adoption as the base model was tested on mobile internet adoption in 2012. We believe that our proposed model can be used to study technology adoption with the addition of health and environment-related variables in the adoption intention of the technology. As we know, all technologies, directly or indirectly, impact our health, and this factor was ignored in the baseline UTAUT2 model.

First, this study shows that five out of six factors are statistically significant for 5G technology adoption. These findings are consistent with previous studies (Alam et al., 2021) mHealth adoption (Shah et al., 2021) 5G purchasing intention (Kim et al., 2007) mobile internet adoption (Alalwan et al., 2018) intention and adoption of internet banking, etc. This means consumers pay attention to IF, PF, SI, HCA, and BI when adopting technology.

Secondly, in SEM results, economic factors (cost value and facilitating conditions) are not statistically significant to 5G technology adoption. This implies that the cost value of 5G technology and associated facilitating conditions that are purely economic factors do not directly affect the 5G adoption. Consumers do not consider these factors when adopting 5G if they are intrinsically motivated, psychologically satisfied, and socially influenced. This is consistent with (Alam et al., 2020). But results at the second stage (*f*sQCA) revealed economic factors are vital for 5G technology adoption. The economic factors in the absence of psychological factors and behavioral intention act as a core condition (primary factor “S6”), whereas in solutions 3, 4, and 5, it appeared as a secondary factor (peripheral condition) affecting 5G technology adoption. At last, it is worth mentioning that “EF” was not absent in any solution. It proves that dual-stage analysis yields a clear picture of consumer psychology.

The third important finding is a negative relationship between intrinsic factors and 5G technology adoption. Consumers’ IF to adopt or reject any technology is associated with their perception of newly introduced technology. As much as the expectations are high in intrinsic factors, the adoption of every new technology is less. This means the threshold level of human psychological and intrinsic perceptions gets higher over time. And it is a continuous phenomenon. Human urge for more advancement and easiness in every technology. They compare benefits with already existing technologies and their decision to adopt or reject them. We also observed that PF also has a negative relationship with BI. It’s simple if newly launched/introduced technology cannot satisfy users’ intrinsic and physiological instincts. Few people will adopt it because they set their threshold very high in terms of expectations and competitive benefits. On the other hand, people will adopt it if newly launched technology has a competitive advantage over contemporary technologies. This is consistent with previous study (Laukkanen et al., 2008).

Further, these findings are also supported in mediation effects. We have observed that BI negatively mediates the relationship between PF and 5G TA. A consumer’s psychological threshold level raises as less likely his BI influences him to adopt the technology. Because he demands more quality and innovation than other available technologies, his satisfaction and expectations are high as they experience different technologies.

Fourthly, the prior researcher has never considered health-consciousness (HC) in technology adoption, specifically 5G technology adoption (Kim et al., 2007; Venkatesh et al., 2012). Our study results revealed that HCA is not only an important independent factor (Shah et al., 2021), but it also plays a significant role as a mediator. Furthermore, IF, PF, SF, and EF positively influence consumers’ HCA to adopt 5G.

Health consciousness attitude (HCA) emerges as an important mediator in technology adoption. It mediates the relationship of IF, PF, and SF in 5G technology adoption. Whenever consumers adopt/buy a newly launched technology, they consider their health before any other factor. HCA does not mediate the relationship between EF and 5G TA. This implies that consumers do not consider economic factors when it comes to their health (Jang et al., 2016). Rejection of H1(d) and H9(d) reflects that consumers do not put their health on the stack.

Fifth, we run *fs*QCA and get six different combinations of our independent variables that provide the same outcome through alternative routes. It is found that BI, HCA, PF, and EF are the core conditions in a different set of configurations to adapt the 5G technology. These findings encourage us to conclude that telecommunication companies and scientists must keep in mind that consumers are not only concerned about their health when adopting or rejecting a new technology, but BI, PF, and EF play a significant role in their decision-making.

Particularly for 5G technology adoption, solutions (1&2) combine PF and BI as main factors with SF and HCA as peripheral conditions regardless of the presence or absence of IF and EF in solution 1 or SF and IF as peripheral factors regardless of the presence or absence of EF and HCA to obtain the same outcome. Solution 3 and 4 lead to the same outcome by incorporating BI as a core factor regardless of the presence or absence of PF. The same outcome is attained after adding BI (core), SF, EF, and HCA as peripheral factors in the absence of IF (solution 3) or solution 4 combining BI (core), IF, SF, and EF as peripheral with the absence of HCA. Solution 5 combines HCA as a core condition with the absence of SF and treats IF, EF, and BI as peripheral conditions, while PF plays a minor role in this solution. Solution 6 comes up with the same output by combining HCA, EF (core factors), IF, and SF (peripheral factors) in the absence of PF and BI. Results indicate that 5G technology adoption can be achieved through these 6 solutions. It also explained that because of the asymmetric relationship between variables (Fiss, 2011), if some variables are insignificant when investigating a direct relationship between explained and exploratory variables, it does not mean that these variables do not influence at all. FsQCA analysis revealed that these variables are still important to make an adoption decision to some extent and can’t be ignored (EF in our case). Hence, we can conclude that a variable can have a core or peripheral role in decision-making, but neglecting its importance in the process is not a good practice.

Finally, the effect of demographic variables integrated as control variables in our model has been checked and found that age, gender, and education have no significant impact on 5G technology adoption. Hence, we can conclude that age, gender, and education influence different technology adoption studies. Still, these variables do not significantly affect 5G technology adoption. Consumers behave alike regardless of different age groups, gender, and education level. The results are similar to recent studies (Dahabiyeh et al., 2021).

## Contribution and Future Recommendations

### Theoretical Contribution

This is the first study to examine consumer attitudes on adopting 5G technology. Our work serves as a pioneer in adopting 5G technology by presenting an empirically validated model at two stages. Our study’s findings make numerous substantial advances to the body of knowledge currently available on technology adoption, particularly 5G technology adoption. Firstly, we have proposed an integrated UTAUT2 model and tested it on 5G adoption. It can be used in future researches to understand and predict consumer technology adoption behavior. Our model explained 77.3% variance in SEM results and 82.9% in *fs*QCA analysis compared to the base model’s 74%.

Second, our proposed technology adoption model provides a complete picture of the user psyche to understand human behavior about technology adoption. It is a valuable contribution to the existing literature. Our methodology can quantify and identify customer intent to adopt newly invented digital technologies.

Thirdly, we have integrated health-consciousness attitude as an independent variable and investigated its mediation role in technology adoption. We have also integrated psychological and environment-related factors in traditional TAM. Furthermore, we have also investigated the mediation role of BI in 5G technology adoption.

Fourthly, we computed a reflective-informative construct for IF, PF, SF, and EF by considering all possible aspects of these factors to study the potential effect of these predictors on technology adoption, specifically 5G. In the existing literature, these factors’ combined effects are rarely investigated.

Additionally, we contributed by our dual-stage methodological approach. The current study analyzed data utilizing a combination of SEM and *fs*QCA, which contributes theoretically to the field of analytical procedures. The *fs*QCA technique can aid in the identification of sufficient causal conditions that contribute to outcomes (Chang and Cheng, 2014). The present study validated the causative relationship between variables using the SEM technique, and the *fs*QCA results suggested six causal configurations for high 5G technology adoption. Results revealed that variance-based models often ignore the importance of some factors (such as an economic factor in our study). Combining the variance-based methods with *fs*QCA can predict a true picture of the consumer’s psyche.

As a whole, our suggested model and methodology are more comprehensive and concrete than previous models since it considers all potential theoretical components of technology adoption, such as psychological elements, economic considerations, intrinsic variables, and social issues and influences. These theoretical conclusions will form the basis for future studies adopting digital technologies, especially 5G technology.

### Practical Implications

This study presents several empirical findings that will be extremely useful to psychiatrists, telecommunication companies, advertising agencies, government officials, and other key decision-makers. We think telecom companies should always consider the economic impact of launching new technologies in a new country while maintaining their current base of customers. Contracts signed with telecommunications firms must account for tax brackets when drafted. New technologies are always leading to new economic opportunities. 5G-related operations include huge economic and revenue production potential (Campbell et al., 2017). Economic activity should be based on ground reality before 5G technology launch in any new locality. Furthermore, people may be less likely to switch to 5G if it is expensive compared to already available 4G (Copeland and Shapiro, 2015).

Secondly, six possible combinations of key factors were identified to gain maximum technology adoption. Companies can choose anyone or the combination of different solutions to attain their goal, keeping in mind the cultural, social, and economic aspects of any 5G market.

Thirdly, our study results found that Health consciousness attitude is a significant factor behind 5G technology adoption (TA). It also serves the significant role of a mediator when it comes to 5G TA. Telecommunications firms’ marketing or advertising departments might target these variables when promoting and launching their product to new markets and growing their customer base in existing markets. They are urged to make such commercials and advertising postings that significantly stir consumer mentality, considering the abovementioned elements. They also support the growth of public knowledge before launching into a new market and promote customer curiosity.

Fourthly, our study’s findings will assist policymakers in developing telecom legislation, as health consciousness is identified as the most critical element influencing 5G technology adoption. Because customers are concerned about their health and the ecological impact of new technology, policymakers must bear that in mind. As long as 5G has a detrimental influence on the environment and human health, people will stop utilizing it. A public awareness campaign can advocate for 5G technology to educate the public about how environmentally friendly 5G technology is. There is no large-scale danger to human health and the ecosystem, and there are no adverse effects.

Lastly, we predict that the telecommunications corporations would form a new market penetration strategy and achieve sizable market shares in other geographic regions. An incentive package tailored to various target market sectors may be developed. In other words, organizations can provide free trial/use of a 5G connection before purchasing, watch for promotional pricing, and so on. The findings of this study may help prepare for 5G expansion.

### Limitations and Future Recommendations

Although we made an effort to address all important parts of our research, our study is limited in several ways. To begin, we drew our sample from a single country. Thus, psychological, cultural, economic, or social aspects may vary in other regions. Therefore, we advocate conducting the same survey across other geographic borders to understand 5G adoption better. Second, we did not collect data on our target population’s income, which may factor in consumers’ 5G adoption decisions. Thus, future research should collect data on the target population’s income and assess its impact on 5G adoption. Third, we did not address demographic characteristics’ mediating or moderating influence. Previous researchers (Venkatesh et al., 2012) examined the moderating effect of age, gender, income, and experience on technology adoption. We propose using these characteristics as moderators to investigate the impact of gender and age on 5G adoption. Finally, because sensitivity analysis demonstrated that HCA is one of the primary drivers of 5G technology adoption, researchers are urged to perform studies on the HCA and ecological implications of 5G technology and the influence of 5G technology waves on ecological systems.

## Data Availability Statement

The original contributions presented in the study are included in the article/Supplementary Material, further inquiries can be directed to the corresponding author/s.

## Author Contributions

SM contributed to conceptualization, methodology, software, and writing – original draft. WZ contributed to supervision and approved the final draft. MS contributed to data collection and analyzing. AA contributed to editing and data collection. GR contributed to visualization and investigation. All authors contributed to the article and approved the submitted version.

## Conflict of Interest

The authors declare that the research was conducted in the absence of any commercial or financial relationships that could be construed as a potential conflict of interest.

## Publisher’s Note

All claims expressed in this article are solely those of the authors and do not necessarily represent those of their affiliated organizations, or those of the publisher, the editors and the reviewers. Any product that may be evaluated in this article, or claim that may be made by its manufacturer, is not guaranteed or endorsed by the publisher.

## Supplementary Material

Supplementary Link: https://drive.google.com/file/d/1PWH1cDkT4aDYE6-TezFPUTbMIKwyCkwN/view?usp=sharing
