# Supplementary material for: Does Health Consciousness Matter to Adopt New Technology? An Integrated Model of UTAUT2 With SEM-fsQCA Approach
Source: Front Psychol. 2022 Feb 10;13:836194. doi: 10.3389/fpsyg.2022.836194 (PMC8868376; doi:10.3389/fpsyg.2022.836194)
Supplement: Supplementary file 1 [file Data_Sheet_1.docx]

**Appendix 1. Measurement items of the Construct**

| **Variables** | **Measurement Items** | | **References** |
| --- | --- | --- | --- |
| Perceived performance | PP1 | I find 5G Internet useful in my daily life. | (Venkatesh et al., 2012) |
|  | PP2 | Using 5G Internet helps me accomplish things more quickly |  |
|  | PP3 | Using 5G Internet increases my productivity. |  |
| Satisfaction | SAT1 | I am satisfied with the speed of 5G internet. | (Jin, Zhou, Lee, & Cheung, 2013) |
|  | SAT2 | I am satisfied with the 5G performance. |  |
|  | SAT3 | I am fully satisfied with 5G services. |  |
|  | SAT4 | I feel satisfied by my experience of using 5G internet. |  |
| Perceived Value | PV1 | The use of the 5G Internet offers value for money. | (H.-W. Kim et al., 2007) |
|  | PV2 | Considering all pros and cons, the use of the 5G Internet is beneficial to me. |  |
|  | PV3 | Despite my familiarity with 5G, the use of 5G Internet is worthwhile to me. |  |
|  | PV4 | Overall, the use of 5G Internet delivers me good value. |  |
| Perceived Functional Value | PFV1 | 5G has an acceptable standard of quality | (H.-W. Kim, Gupta, & Koh, 2011) |
|  | PFV2 | 5G possesses a degree of quality that is satisfactory. |  |
| Social Influence | SI1 | Society members who are influential to me think that I must use 5G Internet | (M. M. D. Alam et al., 2021; Venkatesh et al., 2012) |
|  | SI2 | Society members who influence my behavior think I must use 5G Internet. |  |
|  | SI3 | Society members whose opinions I value prefer that I use 5G Internet. |  |
|  | SI4 | I am inspired by society members who use the 5G internet. |  |
| Habit | HAB1 | It becomes my habit to use 5G internet | (M. M. D. Alam et al., 2021; Venkatesh et al., 2012) |
|  | HAB2 | I am addicted to using 5G Internet. |  |
|  | HAB3 | I must use 5G Internet. |  |
|  | HAB4 | Using 5G Internet has become natural to me. |  |
| Facilitating Condition | FC1 | I have the resources necessary to use 5G Internet. | (M. M. D. Alam et al., 2021; Venkatesh et al., 2012) |
|  | FC2 | I have the knowledge necessary to use 5G Internet. |  |
|  | FC3 | I will use 5G internet if I have a compatible device. |  |
|  | FC4 | 5G Internet is compatible with the devices I use. |  |
|  | FC5 | I can get help from a service provider when I have difficulties using 5G Internet. |  |
| Hedonic Motivation | HM1 | The use of the 5G Internet is fun. | (M. M. D. Alam et al., 2021; Venkatesh et al., 2012) |
|  | HM2 | The use of the 5G Internet is enjoyable. |  |
|  | HM3 | The use of the 5G Internet is very entertaining. |  |
| Cost Value | CV1 | 5G Internet price is reasonable. | (M. M. D. Alam et al., 2021; Venkatesh et al., 2012) |
|  | CV2 | The price I pay for 5G Internet is well-matched of its value. |  |
|  | CV3 | At the present cost, 5G Internet delivers a good value. |  |
| Curiosity | CUR1 | I am curious to use 5G technology. | (Dahabiyeh et al., 2021) |
|  | CUR2 | I want to know more about 5G technology. |  |
|  | CUR3 | I want to know, is 5G works better than 4G technology. |  |
|  | CUR4 | I want to know how much 5G is different from 4G technology. |  |
| Environmental Awareness | EA1 | Before adopting a technology, I consider its environmental effects. | (Shah et al., 2021) |
|  | EA2 | I prefer to use technology that is more environmentally friendly. |  |
|  | EA3 | I am concerned about destroying and wasting the earth's resources. |  |
|  | EA4 | I am aware of 5G radiations and their impact on the environment. |  |
| Environmental Knowledge | EK1 | I am very knowledgeable about 5G radiation and its related environmental issues | (Shah et al., 2021) |
|  | EK2 | I know that I adopt environmentally safe technology. |  |
|  | EK3 | I know that I buy environmentally safe products as I am more cautious about my health. |  |
| Health Consciousness Attitude | HCA1 | I carefully choose 5G technology to ensure good health | (Shah et al., 2021) |
|  | HCA2 | I think I will be a 5G consumer with health consciousness. |  |
|  | HCA3 | I often think about health issues related to different pollution. |  |
| Behavioral intention | BI1 | I intend to continue using 5G Internet in the future. | (Venkatesh et al., 2012) |
|  | BI2 | I plan to continue to use 5G Internet frequently. |  |
|  | BI3 | I will always try to use 5G Internet in my daily life |  |
| 5G Adoption | TA1 | I will use 5G in the future. | (Venkatesh et al., 2012) |
|  | TA2 | I will recommend 5G services to others. |  |
|  | TA3 | 5G internet services increase my willingness to use them. |  |

**Appendix 2. Results of Contrarian case analysis**

| **5G Technology Adoption** | | | | | | | **5G Technology Adoption** | | | | | | |
| --- | --- | --- | --- | --- | --- | --- | --- | --- | --- | --- | --- | --- | --- |
|  |  | **1** | **2** | **3** | **4** | **5** |  |  | **1** | **2** | **3** | **4** | **5** |
| **B.I.** (phi^2^= .75,  p < .001) | 1 | *46 (5.5%)* | *78 (9.4%)* | 40 (4.8%) | **2 (0.24%)** | **0** | **H.C.A.** (phi^2^= .50,  p < .001) | 1 | *48 (5.8%)* | *72 (8.7%)* | 46 (5.5%) | **28 (3.4%)** | **0** |
|  | 2 | *42 (5.0%)* | *22 (2.7%)* | 44 (5.3%) | **32 (3.9%)** | **0** |  | 2 | *18 (2.1%)* | *20 (2.4%)* | 29 (3.5%) | **8 (.96%)** | **8 (.96%)** |
|  | 3 | 60 (7.2%) | 58 (7.0%) | 33 (4.0%) | 18 (2.1%) | 0 |  | 3 | 44 (5.3%) | 42 (5.0%) | 36 (4.3%) | 28 (3.4%) | 6 (.72%) |
|  | 4 | **0** | **6 (.72%)** | 26 (3.1%) | *78 (9.4%)* | *108 (13.0%)* |  | 4 | **38 (4.6%)** | **24 (2.9%)** | 18 (2.1%) | *106 (12.8%)* | *52 (6.3%)* |
|  | 5 | **0** | **0** | 6 (.72%) | *74 (9.0%)* | *57 (6.9%)* |  | 5 | **0** | **6 (.72%)** | 20 (2.4%) | *34 (4.1%)* | *99 (11.9%)* |
| **E.F.** (phi^2^= .36,  p < .001) | 1 | *42 (5.0%)* | *68 (8.2%)* | 36 (4.3%) | **12 (1.4%)** | **0** | **I.F.** (phi^2^= .02,  p < .001) | 1 | *20 (2.4%)* | *38 (4.6%)* | 33 (4.0%) | **37 (4.5%)** | **26 (3.1%)** |
|  | 2 | *44 (5.3%)* | *42 (5.0%)* | 48 (5.8%) | **54 (6.5)** | **10 (1.2%)** |  | 2 | *28 (3.4%)* | *40 (4.8%)* | 30 (3.6%) | **54 (6.5%)** | **37 (4.5%)** |
|  | 3 | 38 (4.6%) | 26 (3.1%) | 28 (3.4%) | 14 (1.7%) | 24 (2.9%) |  | 3 | 26 (3.1%) | 25 (3.0%) | 27 (3.3%) | 29 (3.5%) | 27 (3.3%) |
|  | 4 | **24 (2.9%)** | **20 (2.4%)** | 17 (2.04%) | *60 (7.2%)* | *77 (9.3%)* |  | 4 | **43 (5.2%)** | **39 (4.7%)** | 39 (4.7%) | *45 (5.4%)* | *47 (5.7%)* |
|  | 5 | **0** | **8 (.96%)** | 20 (2.4%) | *64 (7.7%)* | *54 (6.5%)* |  | 5 | **31 (3.7%)** | **22 (2.7%)** | 20 (2.4%) | *39 (4.7%)* | *28 (3.4%)* |
| **P.F.** (phi^2^= .35,  p < .001) | 1 | *48 (5.8%)* | *56 (6.7%)* | 26 (3.1%) | **26 (3.1%)** | **4 (.5%)** | **S.F. (**phi^2^= .26,  p < .001) | 1 | *50 (6.0%)* | *56 (6.7%)* | 29 (3.5%) | **21 (2.5%)** | **4 (.5%)** |
|  | 2 | *36 (4.3%)* | *54 (6.5%)* | 46 (5.5%) | **22 (2.7%)** | **10 (1.2%)** |  | 2 | *43 (5.2%)* | *42 (5.0%)* | 40 (4.8%) | **43 (5.2%)** | **12 (1.4%)** |
|  | 3 | 34 (4.1%) | 36 (4.3%) | 37 (4.5%) | 44 (5.3%) | 26 (3.1%) |  | 3 | 27 (3.3%) | 30 (3.6%) | 31 (3.7%) | 35 (4.2%) | 24 (2.9%) |
|  | 4 | **22 (2.7%)** | **12 (1.4%)** | 26 (3.1%) | *62 (7.5%)* | *34 (4.1%)* |  | 4 | **22 (2.7%)** | **29 (3.5%)** | 34 (4.1%) | *55 (6.6%)* | *48 (5.8%)* |
|  | 5 | **8 (.96%)** | **6 (.72%)** | 14 (1.7%) | *50 (6.0%)* | *91 (11%)* |  | 5 | **6 (.72%)** | **7 (.84%)** | 15 (1.8%) | *50 (6.0%)* | *77 (9.3%)* |
| - Cases in **bold** represent contrarian cases, while cases in *italics* represent the main effect. | | | | | | | | | | | | | |
| - The contrarian cases are counter to the main effect size (phi^2^ range from 0.02 to 0.75). | | | | | | | | | | | | | |
| - BI = Behavioral intention; HCA = Health consciousness attitude; EF = Economic factors; I.F = Intrinsic factors; PF = Psychological Factors; SF = Social factors. | | | | | | | | | | | | | |
