# Supplementary material for: Does Health Consciousness Matter to Adopt New Technology? An Integrated Model of UTAUT2 With SEM-fsQCA Approach
Source: Front Psychol. 2022 Feb 10;13:836194. doi: 10.3389/fpsyg.2022.836194 (PMC8868376; doi:10.3389/fpsyg.2022.836194)
Supplement: Supplementary file 2 [file Data_Sheet_2.docx]

| ***Linearity of relationships*** | | | | | | |
| --- | --- | --- | --- | --- | --- | --- |
|  | | *Sum of Squares* | *df* | *Mean Square* | *F* | *Sig.* |
| Technology Adoption * Intrinsic Factors | (Combined) | 784.104 | 384 | 2.042 | 19.822 | 0.000 |
|  | Linearity | 138.981 | 1 | 138.981 | 1349.156 | 0.000 |
|  | Deviation from Linearity | 645.124 | 383 | 1.684 | 16.351 | 0.000 |
| Technology Adoption * Psychological Factors | (Combined) | 781.520 | 266 | 2.938 | 34.159 | 0.000 |
|  | Linearity | 172.592 | 1 | 172.592 | 2006.611 | 0.000 |
|  | Deviation from Linearity | 608.929 | 265 | 2.298 | 26.716 | 0.000 |
| Technology Adoption * Social Factor | (Combined) | 761.552 | 721 | 1.056 | 1.668 | 0.001 |
|  | Linearity | 180.193 | 1 | 180.193 | 284.547 | 0.000 |
|  | Deviation from Linearity | 581.359 | 720 | 0.807 | 1.275 | 0.057 |
| Technology Adoption * Economic Factors | (Combined) | 778.110 | 236 | 3.297 | 37.719 | 0.000 |
|  | Linearity | 200.011 | 1 | 200.011 | 2288.155 | 0.000 |
|  | Deviation from Linearity | 578.099 | 235 | 2.460 | 28.143 | 0.000 |
| Technology Adoption * Behavioral Intention | (Combined) | 798.584 | 79 | 10.109 | 241.751 | 0.000 |
|  | Linearity | 288.273 | 1 | 288.273 | 6894.108 | 0.000 |
|  | Deviation from Linearity | 510.311 | 78 | 6.542 | 156.464 | 0.000 |
| Technology Adoption * Health Conscious | (Combined) | 551.086 | 32 | 17.221 | 49.220 | 0.000 |
|  | Linearity | 194.489 | 1 | 194.489 | 555.865 | 0.000 |
|  | Deviation from Linearity | 356.597 | 31 | 11.503 | 32.877 | 0.000 |
| Health Conscious * Intrinsic Factors | (Combined) | 787.257 | 384 | 2.050 | 21.347 | 0.000 |
|  | Linearity | 247.038 | 1 | 247.038 | 2572.275 | 0.000 |
|  | Deviation from Linearity | 540.219 | 383 | 1.410 | 14.687 | 0.000 |
| Health Conscious * Psychological Factors | (Combined) | 816.082 | 266 | 3.068 | 124.149 | 0.000 |
|  | Linearity | 307.244 | 1 | 307.244 | 12432.991 | 0.000 |
|  | Deviation from Linearity | 508.838 | 265 | 1.920 | 77.701 | 0.000 |
| Health Conscious *  Social Factor | (Combined) | 740.061 | 721 | 1.026 | 1.233 | 0.087 |
|  | Linearity | 244.556 | 1 | 244.556 | 293.682 | 0.000 |
|  | Deviation from Linearity | 495.505 | 720 | 0.688 | 0.826 | 0.915 |
| Health Conscious * Economic Factors | (Combined) | 788.970 | 236 | 3.343 | 48.323 | 0.000 |
|  | Linearity | 280.337 | 1 | 280.337 | 4052.152 | 0.000 |
|  | Deviation from Linearity | 508.633 | 235 | 2.164 | 31.285 | 0.000 |
| Behavioral Intention * Intrinsic Factors | (Combined) | 802.757 | 384 | 2.091 | 34.206 | 0.000 |
|  | Linearity | 490.651 | 1 | 490.651 | 8028.374 | 0.000 |
|  | Deviation from Linearity | 312.106 | 383 | 0.815 | 13.334 | 0.000 |
| Behavioral Intention * Psychological Factors | (Combined) | 814.594 | 266 | 3.062 | 112.254 | 0.000 |
|  | Linearity | 337.104 | 1 | 337.104 | 12356.780 | 0.000 |
|  | Deviation from Linearity | 477.490 | 265 | 1.802 | 66.048 | 0.000 |
| Behavioral Intention * Social Factor | (Combined) | 784.849 | 721 | 1.089 | 2.607 | 0.000 |
|  | Linearity | 355.708 | 1 | 355.708 | 851.728 | 0.000 |
|  | Deviation from Linearity | 429.141 | 720 | 0.596 | 1.427 | 0.011 |
| Behavioral Intention * Economic Factors | (Combined) | 810.510 | 236 | 3.434 | 104.744 | 0.000 |
|  | Linearity | 516.666 | 1 | 516.666 | 15757.730 | 0.000 |
|  | Deviation from Linearity | 293.843 | 235 | 1.250 | 38.136 | 0.000 |
| Behavioral Intention * Health Conscious | (Combined) | 719.595 | 32 | 22.487 | 162.401 | 0.000 |
|  | Linearity | 411.964 | 1 | 411.964 | 2975.168 | 0.000 |
|  | Deviation from Linearity | 307.630 | 31 | 9.924 | 71.667 | 0.000 |
|  |  | 110.359 | 797 | 0.138 |  |  |
|  |  | 829.953 | 829 |  |  |  |

| ***Cross Loading*** | | | | | | | | | | | | | | | |
| --- | --- | --- | --- | --- | --- | --- | --- | --- | --- | --- | --- | --- | --- | --- | --- |
|  | BI | CUR | CV | EA | EK | FC | HAB | HC | HM | PFV | PP | PS | PV | SI | TA |
| BI1 | 0.893 | 0.479 | 0.541 | 0.640 | 0.070 | 0.749 | 0.402 | 0.726 | 0.462 | 0.524 | 0.683 | 0.494 | 0.239 | 0.421 | 0.539 |
| BI2 | 0.905 | 0.451 | 0.477 | 0.568 | 0.052 | 0.704 | 0.400 | 0.582 | 0.476 | 0.532 | 0.693 | 0.475 | 0.149 | 0.393 | 0.499 |
| BI3 | 0.900 | 0.475 | 0.493 | 0.511 | 0.037 | 0.692 | 0.434 | 0.585 | 0.482 | 0.624 | 0.689 | 0.499 | 0.227 | 0.451 | 0.550 |
| CUR1 | 0.456 | 0.746 | 0.592 | 0.311 | 0.011 | 0.426 | 0.534 | 0.394 | 0.420 | 0.411 | 0.421 | 0.423 | 0.157 | 0.482 | 0.327 |
| CUR2 | 0.412 | 0.849 | 0.511 | 0.286 | 0.014 | 0.361 | 0.487 | 0.322 | 0.425 | 0.322 | 0.343 | 0.352 | 0.101 | 0.483 | 0.317 |
| CUR3 | 0.426 | 0.832 | 0.461 | 0.296 | 0.067 | 0.401 | 0.478 | 0.373 | 0.382 | 0.283 | 0.367 | 0.351 | 0.055 | 0.453 | 0.359 |
| CUR4 | 0.386 | 0.797 | 0.404 | 0.269 | 0.066 | 0.366 | 0.463 | 0.330 | 0.395 | 0.272 | 0.314 | 0.347 | 0.104 | 0.483 | 0.312 |
| CV1 | 0.433 | 0.457 | 0.840 | 0.349 | 0.027 | 0.493 | 0.353 | 0.338 | 0.352 | 0.323 | 0.458 | 0.311 | 0.146 | 0.447 | 0.252 |
| CV2 | 0.464 | 0.529 | 0.879 | 0.329 | 0.011 | 0.483 | 0.473 | 0.306 | 0.406 | 0.343 | 0.442 | 0.273 | 0.128 | 0.438 | 0.285 |
| CV3 | 0.559 | 0.603 | 0.889 | 0.351 | 0.055 | 0.587 | 0.574 | 0.365 | 0.512 | 0.392 | 0.567 | 0.441 | 0.162 | 0.502 | 0.326 |
| EA1 | 0.435 | 0.231 | 0.298 | 0.753 | 0.051 | 0.420 | 0.200 | 0.378 | 0.284 | 0.275 | 0.350 | 0.256 | 0.064 | 0.213 | 0.227 |
| EA2 | 0.428 | 0.303 | 0.315 | 0.753 | 0.089 | 0.417 | 0.213 | 0.442 | 0.258 | 0.267 | 0.338 | 0.271 | -0.021 | 0.235 | 0.243 |
| EA3 | 0.481 | 0.247 | 0.270 | 0.751 | 0.024 | 0.439 | 0.174 | 0.401 | 0.262 | 0.252 | 0.378 | 0.212 | -0.032 | 0.224 | 0.454 |
| EA4 | 0.562 | 0.287 | 0.276 | 0.672 | 0.029 | 0.478 | 0.254 | 0.395 | 0.322 | 0.341 | 0.514 | 0.364 | -0.060 | 0.248 | 0.492 |
| EK1 | 0.062 | 0.058 | 0.056 | 0.082 | 0.872 | 0.056 | 0.001 | 0.102 | 0.017 | 0.007 | 0.071 | 0.062 | 0.003 | 0.009 | 0.058 |
| EK2 | 0.007 | 0.013 | -0.004 | 0.019 | 0.797 | 0.028 | 0.022 | 0.071 | -0.012 | -0.002 | 0.053 | 0.049 | 0.012 | -0.021 | 0.014 |
| EK3 | 0.065 | 0.040 | 0.026 | 0.052 | 0.808 | 0.065 | 0.015 | 0.116 | 0.007 | 0.004 | 0.051 | 0.059 | -0.053 | 0.009 | 0.064 |
| FC1 | 0.705 | 0.395 | 0.521 | 0.525 | 0.072 | 0.877 | 0.371 | 0.528 | 0.459 | 0.456 | 0.646 | 0.468 | 0.225 | 0.336 | 0.490 |
| FC2 | 0.727 | 0.478 | 0.556 | 0.536 | 0.041 | 0.896 | 0.428 | 0.538 | 0.463 | 0.556 | 0.692 | 0.546 | 0.168 | 0.440 | 0.463 |
| FC3 | 0.648 | 0.382 | 0.515 | 0.490 | 0.076 | 0.885 | 0.326 | 0.518 | 0.474 | 0.426 | 0.684 | 0.530 | 0.157 | 0.351 | 0.388 |
| FC4 | 0.742 | 0.454 | 0.542 | 0.544 | 0.035 | 0.890 | 0.372 | 0.548 | 0.471 | 0.461 | 0.701 | 0.514 | 0.154 | 0.398 | 0.447 |
| HAB1 | 0.435 | 0.527 | 0.512 | 0.287 | 0.016 | 0.409 | 0.895 | 0.421 | 0.567 | 0.329 | 0.430 | 0.485 | 0.143 | 0.504 | 0.270 |
| HAB2 | 0.402 | 0.551 | 0.474 | 0.241 | 0.002 | 0.343 | 0.897 | 0.372 | 0.555 | 0.328 | 0.405 | 0.451 | 0.101 | 0.520 | 0.288 |
| HAB3 | 0.374 | 0.536 | 0.446 | 0.215 | 0.017 | 0.365 | 0.853 | 0.315 | 0.508 | 0.375 | 0.396 | 0.413 | 0.137 | 0.468 | 0.191 |
| HC2 | 0.596 | 0.374 | 0.341 | 0.500 | 0.078 | 0.551 | 0.407 | 0.873 | 0.429 | 0.432 | 0.471 | 0.547 | 0.168 | 0.268 | 0.327 |
| HC3 | 0.652 | 0.406 | 0.347 | 0.478 | 0.131 | 0.519 | 0.343 | 0.902 | 0.433 | 0.360 | 0.461 | 0.550 | 0.176 | 0.306 | 0.522 |
| HM1 | 0.424 | 0.504 | 0.441 | 0.313 | 0.048 | 0.435 | 0.517 | 0.399 | 0.861 | 0.347 | 0.450 | 0.480 | 0.105 | 0.396 | 0.303 |
| HM2 | 0.461 | 0.372 | 0.395 | 0.346 | -0.027 | 0.474 | 0.510 | 0.431 | 0.866 | 0.355 | 0.435 | 0.427 | 0.089 | 0.314 | 0.303 |
| HM3 | 0.482 | 0.427 | 0.436 | 0.324 | -0.002 | 0.460 | 0.574 | 0.432 | 0.870 | 0.400 | 0.437 | 0.432 | 0.108 | 0.384 | 0.292 |
| PFV1 | 0.546 | 0.382 | 0.373 | 0.321 | -0.016 | 0.461 | 0.363 | 0.363 | 0.367 | 0.922 | 0.477 | 0.413 | 0.235 | 0.333 | 0.267 |
| PFV2 | 0.608 | 0.363 | 0.382 | 0.380 | 0.022 | 0.531 | 0.359 | 0.456 | 0.418 | 0.934 | 0.544 | 0.392 | 0.265 | 0.323 | 0.329 |
| ﻿PP1 | 0.653 | 0.356 | 0.474 | 0.473 | 0.045 | 0.631 | 0.417 | 0.445 | 0.448 | 0.502 | 0.877 | 0.473 | 0.130 | 0.406 | 0.365 |
| PP2 | 0.655 | 0.419 | 0.490 | 0.433 | 0.079 | 0.660 | 0.415 | 0.464 | 0.446 | 0.475 | 0.877 | 0.497 | 0.193 | 0.410 | 0.370 |
| PP3 | 0.707 | 0.407 | 0.526 | 0.476 | 0.065 | 0.731 | 0.393 | 0.472 | 0.447 | 0.476 | 0.880 | 0.580 | 0.159 | 0.427 | 0.394 |
| SAT1 | 0.403 | 0.285 | 0.219 | 0.252 | 0.036 | 0.373 | 0.351 | 0.477 | 0.307 | 0.337 | 0.400 | 0.691 | 0.091 | 0.275 | 0.259 |
| SAT2 | 0.322 | 0.283 | 0.276 | 0.255 | 0.063 | 0.413 | 0.400 | 0.395 | 0.348 | 0.267 | 0.413 | 0.691 | 0.084 | 0.318 | 0.254 |
| SAT3 | 0.408 | 0.351 | 0.258 | 0.281 | 0.049 | 0.442 | 0.348 | 0.435 | 0.394 | 0.297 | 0.453 | 0.768 | 0.142 | 0.313 | 0.359 |
| SAT4 | 0.469 | 0.419 | 0.402 | 0.291 | 0.057 | 0.479 | 0.411 | 0.517 | 0.462 | 0.376 | 0.470 | 0.801 | 0.099 | 0.354 | 0.367 |
| PV1 | 0.223 | 0.088 | 0.138 | -0.019 | -0.051 | 0.198 | 0.099 | 0.178 | 0.079 | 0.229 | 0.159 | 0.094 | 0.858 | 0.070 | 0.085 |
| PV2 | 0.209 | 0.103 | 0.137 | 0.022 | -0.049 | 0.189 | 0.131 | 0.175 | 0.118 | 0.269 | 0.162 | 0.115 | 0.873 | 0.085 | 0.094 |
| PV3 | 0.149 | 0.124 | 0.149 | -0.048 | 0.009 | 0.121 | 0.142 | 0.143 | 0.099 | 0.208 | 0.135 | 0.124 | 0.862 | 0.154 | 0.086 |
| PV4 | 0.194 | 0.128 | 0.146 | 0.000 | 0.037 | 0.162 | 0.114 | 0.163 | 0.100 | 0.206 | 0.165 | 0.147 | 0.797 | 0.067 | 0.054 |
| SI1 | 0.344 | 0.505 | 0.422 | 0.248 | 0.007 | 0.308 | 0.408 | 0.220 | 0.282 | 0.248 | 0.395 | 0.357 | 0.084 | 0.794 | 0.305 |
| SI2 | 0.379 | 0.463 | 0.428 | 0.234 | 0.028 | 0.346 | 0.461 | 0.285 | 0.370 | 0.266 | 0.361 | 0.331 | 0.135 | 0.808 | 0.253 |
| SI3 | 0.402 | 0.446 | 0.428 | 0.263 | -0.031 | 0.377 | 0.483 | 0.273 | 0.360 | 0.332 | 0.376 | 0.338 | 0.046 | 0.795 | 0.258 |
| SI3 | 0.402 | 0.446 | 0.428 | 0.263 | -0.031 | 0.377 | 0.483 | 0.273 | 0.360 | 0.332 | 0.376 | 0.338 | 0.046 | 0.795 | 0.258 |
| TA1 | 0.412 | 0.267 | 0.278 | 0.387 | 0.073 | 0.433 | 0.214 | 0.411 | 0.304 | 0.151 | 0.305 | 0.331 | 0.066 | 0.229 | 0.749 |
| TA2 | 0.462 | 0.308 | 0.195 | 0.372 | 0.040 | 0.346 | 0.169 | 0.337 | 0.249 | 0.243 | 0.340 | 0.320 | 0.055 | 0.236 | 0.741 |
| TA3 | 0.475 | 0.358 | 0.286 | 0.311 | 0.023 | 0.377 | 0.267 | 0.361 | 0.240 | 0.341 | 0.338 | 0.319 | 0.095 | 0.313 | 0.800 |
